# Development, Optimization and In Vitro/In Vivo Characterization of Collagen-Dextran Spongious Wound Dressings Loaded with Flufenamic Acid

**DOI:** 10.3390/molecules22091552

**Published:** 2017-09-15

**Authors:** Mihaela Violeta Ghica, Mădălina Georgiana Albu Kaya, Cristina-Elena Dinu-Pîrvu, Dumitru Lupuleasa, Denisa Ioana Udeanu

**Affiliations:** 1 Department of Physical and Colloidal Chemistry, Faculty of Pharmacy, University of Medicine and Pharmacy ”Carol Davila”, Bucharest 020956, Romania; mihaela.ghica@umfcd.ro (M.V.G.); ecristinaparvu@yahoo.com (C.-E.D.-P.); 2 Department of Collagen Research, Division of Leather and Footwear Research Institute, National Research & Development Institute for Textiles and Leather, Bucharest 031215, Romania; 3 Department of Pharmaceutical Technology and Biopharmacy, Faculty of Pharmacy, University of Medicine and Pharmacy ”Carol Davila”, Bucharest 020956, Romania; office@colegfarm.ro; 4 Department of Clinical Laboratory and Food Safety, Faculty of Pharmacy, University of Medicine and Pharmacy ”Carol Davila”, Bucharest 020956, Romania; denisaudeanu@gmail.com

**Keywords:** collagen sponges, flufenamic acid, in vitro drug delivery, experimental design, taguchi technique, in vivo burn healing

## Abstract

The aim of this study was the development and optimization of some topical collagen-dextran sponges with flufenamic acid, designed to be potential dressings for burn wounds healing. The sponges were obtained by lyophilization of hydrogels based on type I fibrillar collagen gel extracted from calf hide, dextran and flufenamic acid, crosslinked and un-crosslinked, and designed according to a 3-factor, 3-level Box-Behnken experimental design. The sponges showed good fluid uptake ability quantified by a high swelling ratio. The flufenamic acid release profiles from sponges presented two stages—burst effect resulting in a rapid inflammation reduction, and gradual delivery ensuring the anti-inflammatory effect over a longer burn healing period. The resistance to enzymatic degradation was monitored through a weight loss parameter. The optimization of the sponge formulations was performed based on an experimental design technique combined with response surface methodology, followed by the Taguchi approach to select those formulations that are the least affected by the noise factors. The treatment of experimentally induced burns on animals with selected sponges accelerated the wound healing process and promoted a faster regeneration of the affected epithelial tissues compared to the control group. The results generated by the complex sponge characterization indicate that these formulations could be successfully used for burn dressing applications.

## 1. Introduction

Cutaneous burns are acute wounds characterized by an immediate and increased production of specific proinflammatory mediators both at systemic and local levels, resulting in tissue regeneration delay and other systemic disorders at cardiac, respiratory, renal and intestinal levels [1,2,3,4,5,6]. In addition, tissue injury induces pain signals thus having a direct effect on patients’ quality of life [5,7,8,9]. For these reasons, a first step in burns healing improvement is the control of inflammation and associated pain at the affected tissue.

The use of non-steroidal anti-inflammatory drugs (NSAIDs) ease the post-burn inflammatory response by inhibiting the normal inflammatory phase, successfully reducing both persistent and temporary pain and inflammation near the wound surface without delaying the epithelial healing [7,9,10,11,12,13]. Topical NSAIDs delivery systems offer several benefits compared to the systemic administration route, mainly the avoidance of systemic toxicity (such as gastro-intestinal bleeding) and side effects at hepatic and renal levels, as well as increased patient compliance [7,11,14,15,16]. Also, NSAIDs release directly to the burn level at a sustained rate, ensuring a sufficient and effective drug concentration reaches the affected tissue, which is important in combating and controlling the inflammation and pain that occurs during the healing process [3,9,10].

Among the large group of classical NSAIDs, flufenamic acid (FFA) was selected as the model compound in this research. FFA is an anthranilic acid derivative that inhibits both forms of cyclooxygenase izoenzymes, and is accountable for prostaglandin biosynthesis [17,18,19,20]. FFA demonstrated its anti-inflammatory and analgesic potential in various topical marketed formulations intended for use in different rheumatic disorders and soft tissue injuries [21,22].

NSAIDs-based local burn management currently relies upon the use of biodegradable and non-biodegradable biopolymers as potential carriers for controlled drug delivery systems (also called drug dressings) [23]. Such NSAIDs-based drug delivery systems (DDS) with non-biodegradable polymers support are to be avoided as they need frequent replacements that affect a patient’s comfort and can cause additional trauma to the new epithelial tissue formed, the risk of secondary infection being very high [7,9,24,25,26,27]. Therefore, biodegradable polymers are considered to be ideal vehicles for drugs delivery [28], collagen being preferred due to its properties such as biodegradability, bioresorbability, biocompatibility, hemostasis ability, well-known structure, and reduced manufacturing cost [29,30,31,32,33,34,35]. The use of collagen as drug release support is however limited by its mechanical properties and resistance to in vivo enzymatic degradation, but these characteristics could be enhanced by chemical reaction using various crosslinkers such as glutaraldehyde [31,32,36,37], or by blending with other biopolymers. The biopolymer selected in this study was dextran, a polysaccharide with bacterial origins that has a high tissular biocompatibility and hydrophylicity. Moreover, it increases in vitro stability, decreases in vivo imonogenicity of proteins such as collagen and different enzymes, and is a potential candidate for wound healing stimulation [38,39,40,41].

Collagen can be used in various forms as hydrogels, membranes, fibers, matrices and composite materials, made from undenatured collagen [31,32,33,42]. Due to their ability to absorb large amounts of exudate, to adhere to wet wounds, to maintain a moist healing environment and to favour the regeneration of granular and epithelial tissue at wound level, the porous forms (spongious matrices) obtained by lyophilization of collagen solutions/gels are recommendable for treating burns with different tissue damage degrees [31,42,43]. Collagen spongious matrices have significant potential for burn tissue restoration and can prevent, in association with an anti-inflammatory drug, other further tissue injury caused by the inflammation process [13,44,45,46].

To minimize the overall burn wound inflammation and the unwanted influence of the prolonged inflammatory response, the design of drug controlled delivery systems that ensure both a rapid and gradual drug release is needed. In this context, the proper selection of the drug vehicle is of paramount importance to obtain DDS with desired release characteristics in accordance with the therapeutic indication and application site.

Thus, the aim of this paper was the development, analysis and optimization of some spongious matrices loaded with flufenamic acid, based on a support of collagen and dextran, un- and cross-linked with glutaraldehyde, obtained by lyophilization of hydrogels and designed according to a 3-factor, 3-level Box-Behnken factorial experiment. As drug release from collagen matrices is governed by the combined effect of sponge swelling (and consecutively to drug diffusion through its network) and the hydrolytic activity of the enzymes that exist in the biological fluids (determining the collagen degradation) [30,47,48,49,50], we explored the influence of the formulation factors on the sponges swelling ability and the percent of FFA released from the sponges, as well as on their resistance to enzymatic degradation.

The experimental statistic design was combined with response surface methodology (RSM), this method proving to be efficient in the development and optimization of the formulation of different pharmaceutical systems and technological processes [31,32,51,52,53,54,55,56] as it allows the researcher to investigate the simultaneous effects of formulation factors, to evaluate the interactions effects on system responses, to provide predictive mathematical models, and also to reduce the number of experiments saving in this way both time and resources [52,55,56,57,58,59]. The optimization technique was complemented with the Taguchi approach in order to set—through the Signal-to-Noise indicator (S/N ratio)—the adequate variation conditions for the formulation factors in order to ensure the products’ quality and process robustness [31,32,60,61,62,63].

The selected optimal formulations of the spongious matrices were further subject to preliminary in vivo experiments carried out on Wistar rats in order to test the therapeutic potential in burn healing and to identify any potential major side effect induced by the treatment [64,65,66].

## 2. Results and Discussion

### 2.1. Design of Experiments

A Box-Behnken factorial experiment with 3-factors and 3-levels was used to determine the influence of the formulation factors on some of the physical-chemical and biological sponges parameters considered to be affecting the flufenamic acid delivery mechanism from the designed systems.

The formulation factors (X_i_) selected as independent variables were collagen (C), dextran (DX) and glutaraldehyde (GA) concentrations (g%) (Table 1). The coded levels for each input variable were 1 for low, 2 for middle and 3 for high level respectively. The physical-chemical and biological parameters involved in the drug delivery process from collagen sponges—considered as dependent variables (measured responses—Y_i_)—were swelling ratio, SR (g/g), released percent, RP (%) and weight loss, WL (%) The coded forms of these variables are listed in Table 1 along with the specific constraints imposed by the application of these porous forms in the burn wounds.

Box-Behnken design is a rotatable quadratic design [51,67] where the highest or lowest levels of all factor formulations do not appear simultaneously [68], the trials combinations being placed at the mid-points of each edge and at the center of the cube [51]. Thus, the number of experiments is reduced from 27 (corresponding to the full factorial design) to 13, summarized in Table 2. The spongious matrices were coded as M1 to M13.

### 2.2. Swelling Study

The sponge swelling behavior is an important parameter that needs to be taken into consideration in the formulation of burn dressings. It is a critical parameter for burn healing control, being influenced by the biological properties of the wound and the sponge physical-chemical composition [69].

A swelling analysis was performed to assess the spongious matrices’ absorption medium ability. This is a very important feature as the absorption medium was phosphate buffer pH = 7.4, which was also used as the release medium in the kinetic experiments. The fluid absorption was initiated after sponge immersion in a moist environment and the high retention of fluid within the porous structure could be attributed to the binding of the absorption medium to the collagen and its increased hydrophilicity [9]. These properties also favor the formation of a gel layer after sponge contact with lesion exudate, ensuring the desired wet environment [70].

The fluid absorption characteristics of the designed spongious matrices were quantified using the swelling ratio (SR—g/g), evaluated from the modification of support weight before and after swelling with buffer solution, according to the following formula (Equation (1)):

(1) $$\mathrm{SR}=\frac{\left(W_{t}-W_{0}\right)}{W_{0}}$$

where W_0_ is the dried sponge weight at the initial time, and W_t_ is the sponge weight after immersion at time t [31].

The swelling ratios of the collagen spongious matrices following 10 h of immersion in phosphate buffer pH 7.4 are summarized in Table 2. The designed sponges exhibited a high swelling ability, the values recorded for the swelling ratio being between 24.05 and 45.65 g/g, proving their capacity for absorbing a large amount of burn exudate, adhering to the wet lesion and favoring the formation of new regenerated tissue. A high absorption capacity is also a valuable property required in sponge design as drug diffusion depends on fluid penetration of the porous structure [31].

### 2.3. In Vitro Drug Release Kinetics Study and Data Modeling

**Figure 1:**
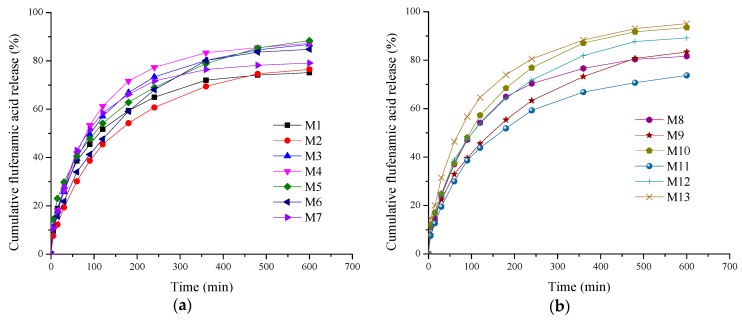
Time-dependent cumulative release profiles of FFA from collagen sponges: (**a**) experiments 1 to 7; (**b**) experiments 8 to 13.

The cumulative percentage of flufenamic acid released throughout the in vitro experiments is graphically presented in Figure 1a,b.

The charts presented in Figure 1 indicate that the designed formulations present similar kinetic profiles. The flufenamic acid is markedly released in the first 60 min and the released drug percentage ranges from 29.97% (M11) to 46.37% (M13). This behavior—known as burst effect—is followed by a progressive anti-inflammatory drug delivery over a longer period of time set to 9 h. At the end of 10 h, a stationary drug release is reached.

This biphasic pattern of release involving the two stages is targeted to control the local inflammation and pain associated with a burn injury. Thus, the burst release effect ensures both a rapid reduction of painful sensation and the management of the pro-inflammatory mediators’ cascade released at the burn level, and is needed immediately after lesion occurrence. The gradual drug delivery phase offers an anti-inflammatory and analgesic local effect over the longer period needed for burn healing.

The drug is released at a cumulative percentage higher than 73.69 and attaining 95.09% after 10 h of experiments (Table 2), resulting in an advantage for burn lesions treatment. This release profile is desirable for burn treatment as the first 12 h are critical and correspond to the peak of the inflammatory phase [70].

The flufenamic acid release profiles from the spongious matrices were assessed to determine the kinetic mechanism.

The drug mass transfer mechanism was evaluated by fitting the in vitro release experimental data to the Power law model (Equation (2)) and its particular case, the Higuchi kinetic model (Equation (3)) [71,72,73]:

(2) $$\;\frac{m_{t}}{m_{\infty }}=k\;\cdot \;t^{n}$$

(3) $$\;\frac{m_{t}}{m_{\infty }}=k\;\cdot \;t^{0.5}$$

where m_t_/m_∞_ is the fractional drug released in time t, k—the kinetic constant—and n—the release exponent characteristic—to the drug transport mechanism.

As noticed in Table 3, the values recorded for the determination coefficients (R^2^) are higher for the Power law model (superior to 0.95) indicating a non-Fickian mechanism for FFA release from collagen spongious matrices, in line with our previous studies. The release exponent and kinetic constant values specific to the Power law model are listed in Table 3.

As we previously reported, and in accordance with other authors, this mechanism can be considered specific to drug delivery from swellable hydrophilic matrices like sponges and depends on many processes. Thus, a burn wound initially has a high content of exudate absorbed by, and penetrating, the porous spongious structure. A gel layer is formed at the contact between the wound and the sponge dressing, favoring the rapid diffusion of the drug retained at, and close to, the surface. The sponge’s higher capacity for absorbing a large amount of fluid immediately after contact with the “fresh” wound is correlated with the drug’s fast release in the first 60 min of experiments, reducing the burn inflammation and the associated pain at the application site.

The absorption process is followed by the hydration of the polymer network and simultaneous swelling of the relaxed polymer, and diffusion through the swollen matrix of drug entrapped in the polymer network during the lyophilization process.

The complex drug release mechanism from spongious forms previously described determines the non-respect of the Higuchi kinetic profiles characterized by a smaller drug diffusion rate than polymer relaxation rate.

Currently, it is considered that the potential erosion of the spongious support could also affect the drug delivery [47,48,49,50]. Therefore, an enzymatic study is required as a further step in tailoring the drug release kinetics.

### 2.4. In Vitro Enzymatic Degradation Analysis

In vitro biodegradation of collagen spongious matrices by collagenase solution was needed to simulate the in vivo behavior of the designed wound dressings. The degradation rate control is an important aspect in burn healing as in vivo resorption influences the tissue regeneration capacity [42]. Thus, a high degradation rate induces a very fast drug release due to the rapid support destructuring, which results in a loss of treatment efficiency, while a small degradation rate leads to a delay in affected tissue healing due to the necessity to successively remove and replace a wound dressing. Also, in this last case there is the possibility of producing new lesions and additional suffering, affecting patients’ comfort.

Enzymatic degradation of collagen materials is dependent on, and determined by, their triple helical integrity, the crosslinking degree, and the availability of cleavage sites [74].

The resistance to enzymatic degradation was monitored through a weight loss parameter determined using the following equation (Equation (4)):

(4) $$\%\;\mathrm{Weight}\;\mathrm{Loss}=\frac{W_{i}-W_{t}}{W_{i}}\;\times \;100$$

where W_i_ is the sponge initial weight, and W_t_—the weight of the samples after a time t [75].

The weight loss values up to 10 h varied from 21.48 to 38.02 (%) as indicated in Table 2.

### 2.5. Optimization Technique

The experiments resulting from the Box-Behnken factorial design were subjected to an optimization technique based on experimental design, response surface methodology, and the Taguchi approach—presented in our previous works [31,32,60]—in order to determine the influence of the formulation factors on some responses involved in drug delivery from collagen spongious matrices. The physical levels of the independent variables to be optimized are considered for the hydrogels lyophilized to obtain the spongious matrices.

In order to set the reduced quadratic polynomial equations for each response, a stepwise regression analysis with backward elimination subroutine—which automatically eliminated the insignificant terms—was applied to the experimental data obtained. These regression models showing the quantitative effect of the formulation factors (X_i_) and their interactions on the responses (Y_i_, Table 2) are presented as Equations (5)–(7), with only the significant terms (*p* < 0.05) being kept. The interactions between the formulation factors are the result of the terms containing two such parameters while each variable square indicate the quadratic relationship. The coefficients value for each independent variable, the combination of two independent variables, or the squared independent variables show their effects on each response. For the responses to be maximized—the swelling ratio and released drug percentage—the coefficients’ positive sign signifies a synergistic effect and their negative sign signifies an antagonist effect. For the response to be minimized—weight loss—the signs’ meaning is reversed.

From Equation (5) a positive impact on Y_1_ response is noticed for X_2_ (quadratic effect), as well as for the interactions between X_1_ and X_2_ and the interaction between X_1_ and X_3_, while X_1_ (quadratic effect), X_2_ (linear effect), X_3_ (quadratic effect) and the interaction between X_2_ and X_3_ all have an antagonist effect on this response. Equation (6) indicates that the released drug percentage is positively influenced by X_1_ and X_2_ (linear effect) and by the interaction between X_1_ and X_3_, and negatively influenced by the quadratic effect of all formulation factors. Also, a positive quadratic effect of variables X_1_ and X_3_ and also of the interaction between X_1_ and X_2_ on the resistance to enzymatic degradation can be observed from Equation (7).

It can be noticed that the resistance to enzymatic degradation is influenced by the smallest number of factors.

The above equations presented in terms of coded factors are given below:

(5) $$Y_{1}=59.58-39.22X_{2}-23.45X_{1}^{2}+13.92X_{2}^{2}-68353.76X_{3}^{2}+27.70X_{1}X_{2}+772.57X_{1}X_{3}-775.26X_{2}X_{3}$$

(6) $$Y_{2}=-105.15+379.47X_{1}+30.77X_{2}-192.21X_{1}^{2}-17.29X_{2}^{2}-120827.46X_{3}^{2}+905.61X_{1}X_{3}$$

(7) $$Y_{3}=43.15-6.75X_{1}^{2}-57253.15X_{3}^{2}-3.36X_{1}X_{2}$$

The predictive power of the regression models obtained was evaluated by way of goodness of fit (correlation—R, and determination—R^2^ coefficients), analysis of variance (ANOVA) and residual analysis. The correlation coefficients values are 0.9951, 0.9899 and 0.9740 respectively, showing a high correlation degree between the observed and predicted values. The determination coefficients are 0.9902, 0.9799 and 0.9487 and indicate that only 0.98%, 2.01% and 5.13% of the total system response variation is not explained by the corresponding model.

The summary of variance analysis is presented in Table 4.

It can be seen that the application of ANOVA proves the statistical significance of each regression model.

**Figure 2:**
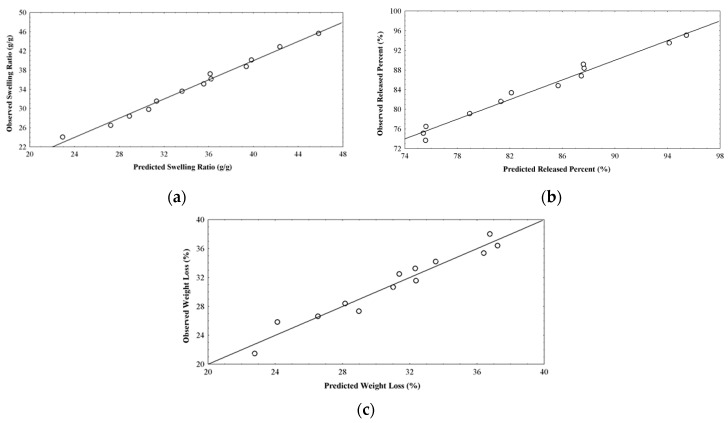
Plot showing correlation between observed and predicted values for: (**a**) swelling ratio (g/g); (**b**) released percent (%); (**c**) weight loss (%).

The good correlation between observed and predicted values is also sustained by the residual analysis presented in Table 2 and Figure 2, in which a linear distribution is noticed.

**Figure 3:**
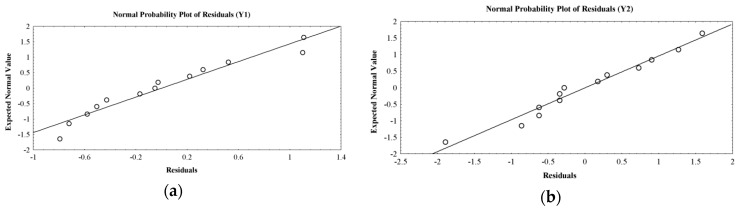

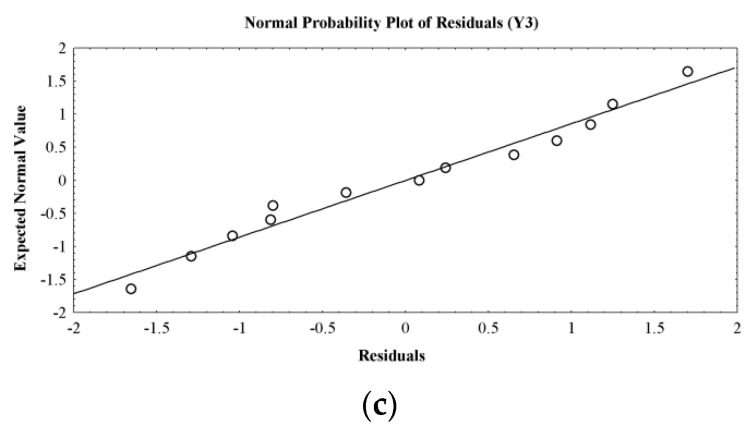
Plot showing correlation between expected normal values and residuals for: (**a**) swelling ratio (g/g); (**b**) released percent (%); (**c**) weight loss (%).

Moreover, the design normality was validated by normal probability plots of residuals (difference between observed and predicted responses). The experimental values for the responses are distributed near a straight line, confirming the robustness of design (Figure 3).

All statistical tests performed highlighted the high predictive power of the selected reduced regression polynomial models and the possibility of their use in correlating and validating the experimental results.

The influence of the formulation factors on sponge responses can be further visualized by plotting three-dimensional surface graphs. This technique—known as response surface methodology—is applied in order to assess the relationship between various independent variable combinations and for the determination of the conditions needed to obtain the targeted drug delivery kinetics.

**Figure 4:**
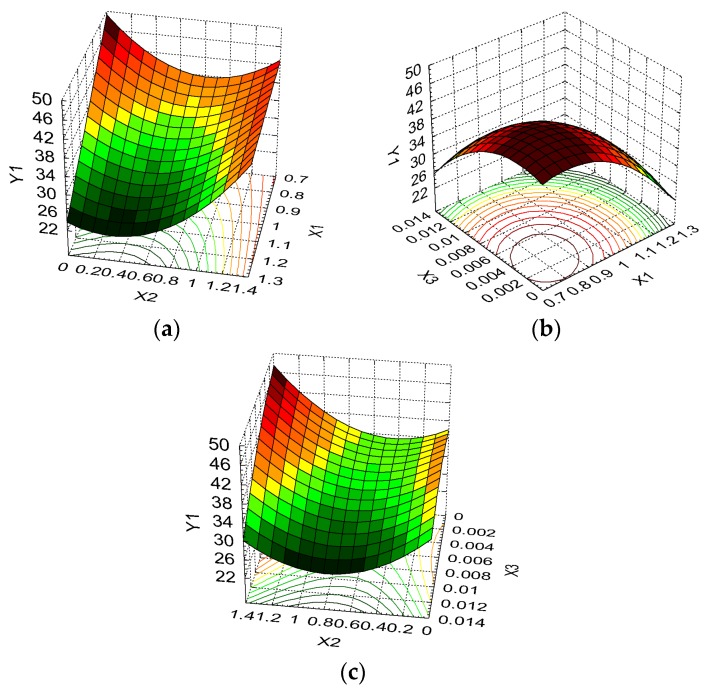
3D response surface and contour plot showing the effect of various formulation factors on swelling ratio (Y_1_): (**a**) collagen concentration (X_1_) and dextran concentration (X_2_); (**b**) collagen concentration (X_1_) and glutaraldehyde concentration (X_3_); (**c**) dextran concentration (X_2_) and glutaraldehyde concentration (X_3_).

**Figure 5:**
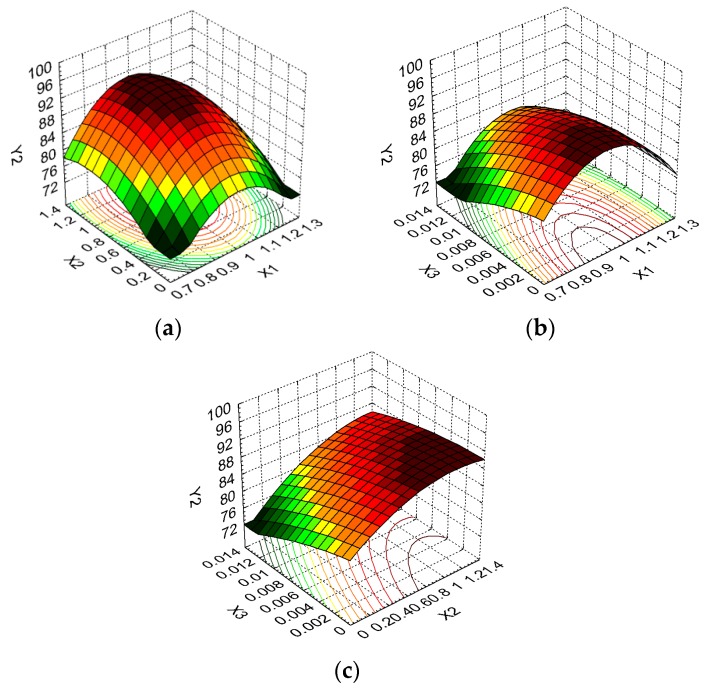
3D response surface and contour plot showing the effect of various formulation factors on released percent (Y_2_): (**a**) collagen concentration (X_1_) and dextran concentration (X_2_); (**b**) collagen concentration (X_1_) and glutaraldehyde concentration (X_3_); (**c**) dextran concentration (X_2_) and glutaraldehyde concentration (X_3_).

**Figure 6:**
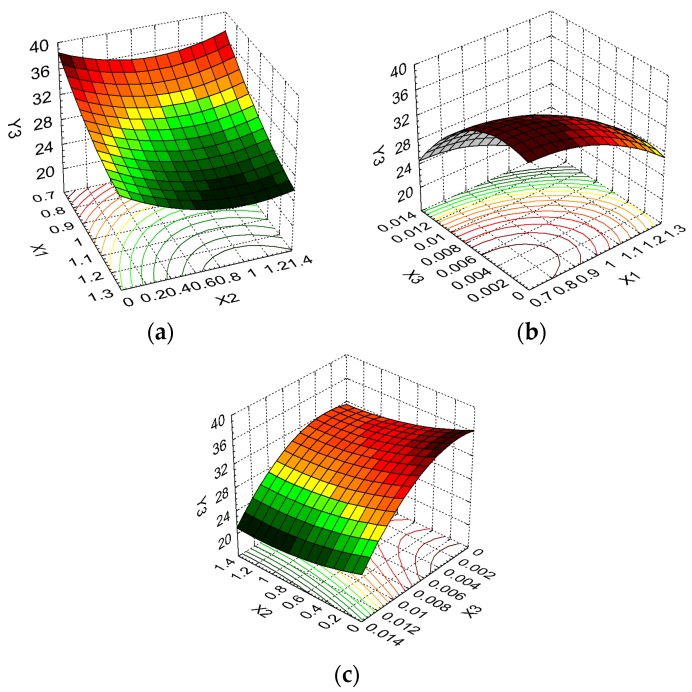
3D response surface and contour plot showing the effect of of various formulation factors on weight loss (Y_3_): (**a**) collagen concentration (X_1_) and dextran concentration (X_2_); (**b**) collagen concentration (X_1_) and glutaraldehyde concentration (X_3_); (**c**) dextran concentration (X_2_) and glutaraldehyde concentration (X_3_).

The response surfaces are presented in Figure 4, Figure 5 and Figure 6.

From Figure 4a we can see that the swelling ratio is strongly influenced by the collagen content for small amounts of dextran, this variation being attenuated by the addition of dextran. The swelling ratio decreases from 45.65 g/g (maximum observed value) to 28.41 g/g when collagen content increases and dextran is not present in the formulation (37.76% decrease) and from 31.56 g/g to 24.05 g/g (23.80% decrease) for dextran at the maximum level. It is suggested that the addition of collagen to the formulation increases the number of sponge pores, favoring the swelling process.

Figure 4b shows the dependence of the swelling ratio on the concentration of collagen and glutaraldehyde. A high swelling ratio is favored by both formulation factors at the lowest level. Y_1_ has a more pronounced increase from 26.52 g/g to 38.77 g/g (46.19%) with a decrease in collagen concentration when the glutaraldehyde level is kept at a minimum, while the increase recorded for Y_1_ is from 31.56 g/g to 38.77 g/g (22.84%) when the glutaraldehyde concentration decreases and the collagen level is kept at a minimum.

Concerning the dextran and glutaraldehyde influence on the swelling ratio, one can conclude from Figure 4c that the variation pattern is similar for different concentrations of glutaraldehyde, but small concentrations of glutaraldehyde and high quantities of dextran favor high values of Y_1_. This variation is exemplified by a Y_1_ increase of 15.17% (from 37.23 g/g to 42.88 g/g) when no glutaraldehyde is present in the formulation and dextran varies from the minimum to maximum level. Also, the smallest values for the swelling ratio are the result of dextran at the middle level and glutaraldehyde at the maximum level.

Figure 5a highlights the dependence of released drug percentage on collagen and dextran concentrations. The collagen variation influences Y_2_ response, the highest released drug percentage being reached for mid to high amounts of dextran and mid values of collagen. Y_2_ displays a more important variation from 73.69% to 95.09% (29.04% increase) with dextran concentration increase when collagen is at the middle level. Similar evolutions, but with smaller Y_2_ increases, are seen when collagen is at the low and high levels and dextran varies between low to high levels.

The same variation pattern is also recorded for the influence of collagen and glutaraldehyde concentrations on released drug percentage, as it results from Figure 5b. The glutaraldehyde crosslinking properties are obvious as its absence results in high released drug percentage when collagen is at the middle level, the addition of this substance involving a reduction for Y_2_ values.

Figure 5c shows the improvement of released drug percentage with the addition of dextran in the presence of glutaraldehyde. The drug release is favored by high amounts of dextran and by the lack of glutaraldehyde, as already mentioned. The released drug percentage increases by 26.90%—from 73.69%, in the absence of dextran and glutaraldehyde at the high level, to 93.51% when there is no glutaraldehyde but the maximum amount of dextran is included in the formulation.

According to the shape of Figure 6a, the upper level of collagen and the middle level of dextran determine a small weight loss value, the collagen variation having more influence on the Y_3_ value. Thus, the Y_3_ value decreases from 31.37% to 26.53% (15.43% decrease) for a dextran variation from minimum to maximum level at a maximum level of collagen and from 27.32% to 21.48% (21.38% decrease) for a collagen variation from minimum to maximum level for a middle level of dextran.

From Figure 6b we can conclude that the resistance to enzymatic degradation is increased for high collagen and glutaraldehyde values, both parameters having a similar effect on Y_3_. This suggests that a higher concentration of collagen induces a denser matrix that is resistant to enzymatic degradation. Y_3_ values decrease from 36.42% to 21.48% when collagen is at a minimum level and glutaraldehyde is absent from the formulation. When collagen and glutaraldehyde are at maximum levels, Y_3_ values decrease by 41.02%.

According to Figure 6c, the analysis of dextran and glutaraldehyde formulation parameters indicates that the former has a small influence, and the latter an important influence, on Y_3_ response, underlining the importance of glutaraldehyde concentration for resistance to enzymatic degradation.

As the hypothesis of this study is that drug release is controlled by both swelling degree and drug diffusion through the swollen polymeric matrix, as well as by the sponge support degradation rate, it was noticed that a small to medium degree of cross-linking determined higher values for the swelling ratio and released drug percentage simultaneously, with enhanced mobility of the polymeric chains resulting in macromolecular mobility and favoring drug diffusion. Meanwhile, a higher crosslinking degree is related to the incorporation of the drug into the sponge structure, reducing the polymeric chain hydration and consequently restricts the drug diffusion through the network matrix, but at the same time increases the resistance to enzymatic degradation.

In order to determine the sponge formulations that lead to responses that are minimally affected by noise factors, the final stage of our research involved the assessment of the effect of control factors on each response using the Signal-to-Noise ratio (S/N ratio), a performance indicator proposed by Taguchi.

The signal represents the desired value of the control factor (independent variable) while the noise represents the unwanted influence, the selection of a defined S/N ratio [32,60] depending on the constraints imposed on system responses. Thus, taking into consideration the constraints mentioned in Table 1b, the “larger-the-better” criterion has to be applied for S/N ratios related to swelling ability and the percentage of FFA released from the sponges, and the “smaller-the-better” criterion for the weight loss, which reflects resistance to enzymatic degradation.

The control factors’ influence on the S/N ratio for each response, resulting in the optimal combination of formulation factors, is given in Table 5.

**Figure 7:**
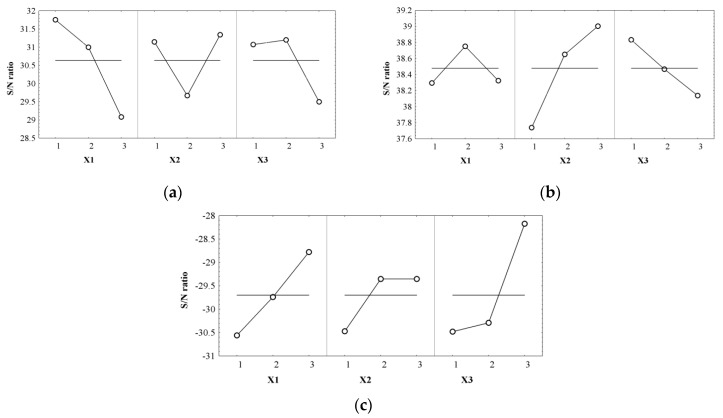
Control factors effects on S/N ratio for: (**a**) swelling ratio (Y_1_); (**b**) released percent (Y_2_); (**c**) weight loss (Y_3_).

The responses variation when the formulation factors are modified from low to high level is illustrated in Figure 7.

From Figure 7, and taking into account the differences between the variation levels for all responses, we can conclude that collagen has a primary influence on the swelling ratio and a moderate influence on released drug percentage and weight loss. The optimum coded level for this formulation factor are 1 for swelling ratio (corresponding to a X_1_ concentration of 0.8), 2 for released drug percentage (corresponding to a X_1_ concentration of 1.0) and 3 for the weight loss (corresponding to a X_1_ concentration of 1.2).

The dextran has a primary influence on released drug percentage, a moderate influence on the swelling ratio, and a small influence on resistance to enzymatic degradation. The optimum coded levels of dextran for reducing noise factors to a minimum extent are 3 (maximum concentration of dextran, 1.2) for Y_1_ and Y_2_ responses, and 2 for resistance to enzymatic degradation.

The most important difference detected in the variation levels for glutaraldehyde is for the weight loss, while the other formulation factors are moderately affected by X_3_. The reduction of noise factor effects occurs for the following glutaraldehyde optimum coded levels: 2 for Y_1_ (middle level of X_1_), 1 for Y_2_ (absence of glutaraldehyde from the formulation) and 3 for Y_3_ (maximum level of glutaraldehyde).

Following the analysis of the formulation factors’ effect size on the responses, the results show that the main variable affecting the swelling ratio is collagen concentration (effect size 1.58 times higher than for the dextran concentration and 1.97 times higher than for the glutaraldehyde concentration).

The main effect of dextran is on the released drug percent, the effect size being 1.92 times higher in comparison with collagen concentration and 1.48 times higher than the glutaraldehyde concentration.

The glutaraldehyde strongly influences resistance to enzymatic degradation, and the collagen also has an important effect on this response. Thus, the glutaraldehyde effect size for the weight loss is 1.66 times superior to that of collagen concentration and is 4.49 times superior to that of dextran concentration.

By application of the Taguchi technique, three optimal stable and robust formulations were selected that least affect the swelling ratio, released drug percentage and sponge weight loss, see Table 6 for their compositions. All these formulations are included in the experimental design, their compositions being coded as 1:3:2 (sponge M3) for swelling ratio, 2:3:1 for released drug percentage (sponge M10) and 3:2:3 (sponge M8) for the weight loss which expresses resistance to enzymatic degradation.

The regression models used proved to have strong predictive power according to the values presented in Table 6, the errors recorded being in the range (−5.67 ÷ 4.84)%.

It can be remarked that the best combination of formulation factors is not necessarily made by the highest/smallest values for the responses, but by the combination that leads to responses that are stable, robust, and insensitive to the noise factors, because the selection of an optimal product or of its manufacturing conditions consists not only in obtaining the best values of the responses, but also in finding the conditions for which the characteristics vary to the minimum extent [60].

### 2.6. Evaluation of the Collagen Sponges’ Performance during the Wound Healing Process

The burn healing and anti-inflammatory action of the optimal sponges previously selected was confirmed by in vivo experiments performed on Wistar rats.

The animals were distributed in 4 groups of 5 individuals each as follows: M3 group was treated with the M3 FFA-sponge, M8 group with the M8 FFA-sponge, M10 group with the M10 FFA-sponge, and the Control group was not treated. M3 is a cross-linked sponge with a smaller concentration of collagen, but with a higher dextran concentration, M8 is a cross-linked sponge with a higher concentration of GA and a medium dextran concentration, and M10 is an uncross-linked sponge with a medium collagen concentration and a higher dextran concentration (Table 6).

**Figure 8:**
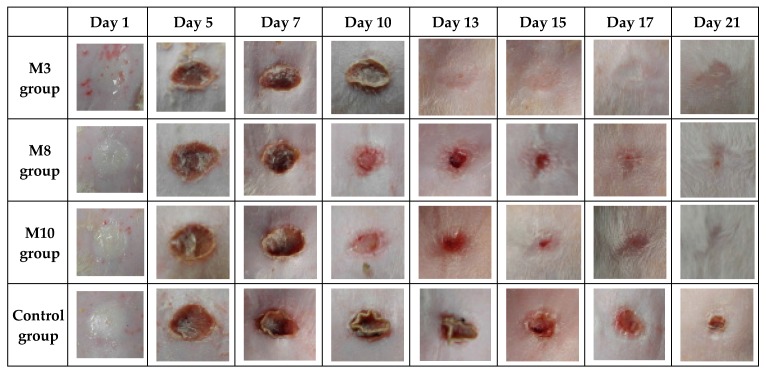
Evolution of wound healing process in rats without treatment (Control) and treated with collagen sponges M3, M8, M10 at different time intervals (day 1 is considered t = 0).

The macroscopic images of burn wounds experimentally induced in rats are presented in Figure 8. The wounds were initially characterized by a white eschar with an affected epidermis and dermis and a hyperaemic zone in the periphery. During the first day, the wound area became fully hyperaemic due to red blood cell extravasation and a post-traumatic inflammatory process.

After inducing the burns, the application of the sponges accelerated the healing process of the lesions in comparison with the control group. After 24 h, the sponges were completely absorbed at the wound area and the epithelial tissue had started to cicatrize. 

The healing process was evaluated according to the size profile of the wound as described by the following equation (Equation (8)):

(8) $$\mathrm{Healing}\;\%=\frac{W_{i}-W_{t}}{W_{i}}\;\times 100$$

where the wound size (W) is an average measurement from the longest and shortest dimensions of the affected tissue, W_i_ is the initial wound size and W_t_ is the wound size at different time intervals [13,27,64,65,66]. The wound was considered healed when the scab fell off the experimental lesion.

**Figure 9:**
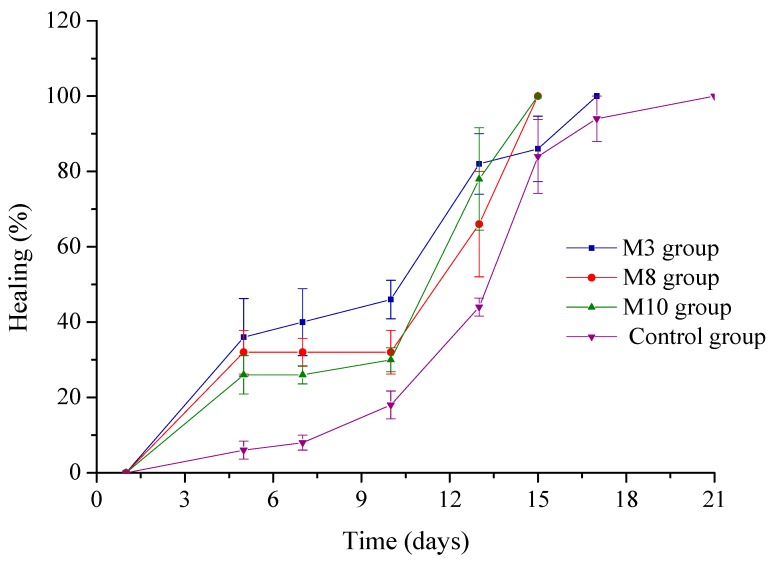
Evolution of healing process after treatment with collagen sponges (M3, M8, M10) and not-treated control group.

The evolution of the wound diameter (mm) is presented in Table 7. The healing process was calculated according to Equation (8) and is presented in Figure 9.

The wound size was reduced by 36% in the case of treatment with the M3 sponge after 5 days, followed by the groups treated with the M8 and M10, which showed a decrease in wound size of 32% and 26% respectively in comparison with the control group (Table 7, Figure 9, ANOVA *p* < 0.05). After 7 days, the wound diameter in case of the treated groups decreased significantly compared to the mean diameter of the control group (*p* < 0.05), corresponding to a faster healing process. In the case of some animals from groups treated with M8 and M10 sponges, the scab fell off after 10 days and the healing process was completed after 15 days from the start of treatment. In the case of M3 the wound mean diameter was slightly decreased in the first 10 days in comparison with M8 and M10 groups (*p* > 0.05) but healing was completed for all animals after 17 days. The first 10 days after a burn are critical for tissue regeneration and the results demonstrated a significantly improved healing process after comparison of the treated groups with the control group (*p* < 0.05, Table 7).

Our preliminary pharmacological study identified no significant differences in the healing process after treated groups comparison (*p* > 0.05). We observed that in case of the M3 group, after the scab fell off, the scar left was less visible compared to other groups (Figure 8). Further biological studies will be required to elucidate the skin repair mechanism and to explain how this cross-linked sponge with a smaller collagen concentration and a higher dextran concentration may offer better support for epithelial regeneration.

## 3. Materials and Methods

### 3.1. Materials

Type I collagen gel with a concentration of 2.54% (*w*/*w*) and pH 2.5 was extracted from calf hide by a technology currently used at the Collagen Department of Division Leather and Footwear Research Institute, Bucharest, Romania [42]. The chemicals were purchased from commercial suppliers as follows: flufenamic acid from MP Biomedicals (Solon, OH, USA), dextran from *Leuconostoc* spp. (Mw = 15,000–25,000) from Fluka (Steinheim, Germany), glutaraldehyde from Merck (Darmstadt, Germany), type I collagenase obtained from *Clostridium histolyticum* from Sigma-Aldrich (St. Louis, MO, USA), sodium hydroxide, monobasic potassium phosphate and disodium hydrogen phosphate from Merck (Germany), ether ethylic from Sigma Aldrich (USA). The water used was distilled and all other chemicals used for analysis were of analytical grade. The animals used for the pharmacological experiments were Wistar male rats and were supplied by the Animal Biobase of the “Carol Davila” University of Medicine and Pharmacy, Bucharest, Romania.

### 3.2. Methods

#### 3.2.1. Preparation of Spongious Matrices

**Figure 10:**
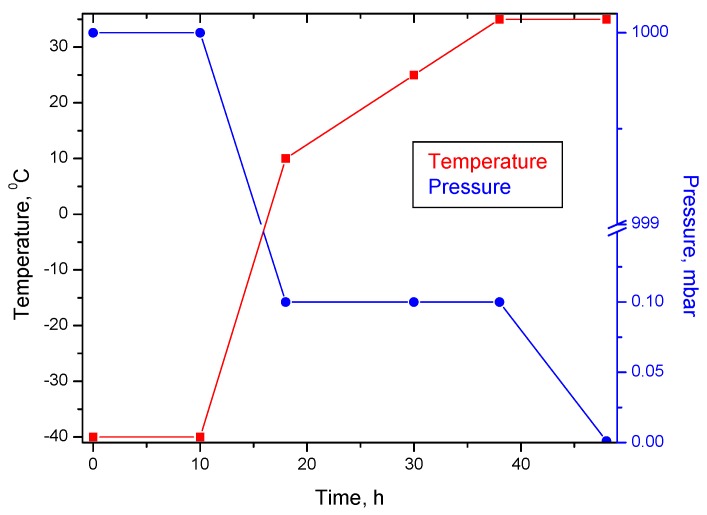
Freeze-drying chart of collagen-flufenamic acid spongious matrices.

Collagen gels with concentrations of 0.8%, 1.0% and 1.2% and pH 7.4 were obtained from the initial collagen gel (2.54% and pH 2.5) using an NaOH 1 M solution under mechanical stirring. 0.6% and 1.2% dextran and 0.5% flufenamic acid solutions were added to the collagen gels according to the compositions presented in Table 1. Some of the hydrogels were cross-linked with 0.006% and 0.012% glutaraldehyde for 24 h at 4 °C. The amounts of dextran, flufenamic acid and glutaraldehyde were reported to collagen dry substance. The hydrogels were designed according to the 3^3^ Box-Behnken design and their final composition is presented in Table 2. The hydrogels were then lyophilized using the Delta LSC 2-24 Martin Christ lyophilizer (Osterode am Harz, Germany) by the method previously described [37] and following the program presented in Figure 10. In this way, the corresponding spongious matrices were obtained and coded as M1 to M13. Briefly, the process started with freezing at −40 °C for 10 h and continued with main freeze drying at 0.1 mbar and the temperatures of +10 °C for 8 h, +25 °C for 12 h and +35 °C for 8 h. The final freeze drying lasted 10 h at the pressure of 0.001 mbar and the temperature of 35 °C.

#### 3.2.2. Swelling Study

The spongious matrices’ swelling ability was assessed using a general gravimetric method, at 37 °C. Collagen sponges were carefully weighed when in a dry condition (W_0_) and were then immersed in the absorption medium represented by phosphate buffer solution pH 7.4 which simulates the wound physiological liquid pH [31]. At specific time intervals over a 10 h period, the swollen sponges were taken out of the swelling medium, hung to dry for one minute until no more drops were formed, then weighed (W_t_) with a four decimal point electronic microbalance, and the swelling ratio was determined. Each experiment was conducted in triplicate and the results were averaged.

#### 3.2.3. In Vitro Drug Release Kinetics Study and Data Modeling

The studies of flufenamic acid in vitro release kinetics from the collagen sponges were performed using dissolution equipment in conjunction with paddle stirrers (Essa Dissolver, city, Italy), as previously reported [76]. The disc shaped sponge samples had a diameter of 3 cm and a predetermined weight, and were put in a transdermal sandwich device before being placed in apparatus dissolution vessels. The determinations were carried out at 37 °C ± 0.5 °C with a rotational speed of 50 rpm. The release medium used was a phosphate buffer solution of pH 7.4. At pre-established time intervals over a 10 h period, samples of 5 mL were collected from the receiving medium and replaced with an equal volume of fresh phosphate buffer solution, maintained at 37 °C ± 0.5 °C, to keep a constant volume in the release vessel. The concentration of FFA in each sample was monitored by UV spectroscopy (Perkin-Elmer UV-Vis spectrophotometer, Überlingen, Germany) at its maximum absorbance corresponding to a wavelength of 288 nm, using the standard curve $(A_{1\%}^{1\mathrm{cm}}=534$), and the cumulative released drug percentage was determined. The release studies were performed in triplicate.

#### 3.2.4. In Vitro Enzymatic Degradation Analysis

The enzymatic degradation of spongious matrices was investigated in a phosphate buffer solution with pH 7.4 containing 10^−6^ mg/mL collagenase and quantified using weight loss (WL) as a function of time. Sponge samples of 1 cm × 1 cm, accurately weighed (W_i_), were immersed in the collagenase solution and incubated at 37 °C. At fixed time intervals over a 10 h period, the sponge pieces were extracted, weighed (W_t_) and the degradation percentage was evaluated. Three determinations were performed for each sample and the results were averaged.

#### 3.2.5. Design of Experiments and Optimization Technique

The preparation of the collagen hydrogels used to obtain the corresponding spongious matrices through lyophilization was performed in accordance with a 3-factor, 3-level Box-Behnken factorial design (Table 1 and Table 2). The experiments resulting from the Box-Behnken factorial design were performed randomly and in triplicate to minimize the errors due to systematic trends in the factors. The statistical data analysis was performed using different routines of Statistica StatSoft Release software package. In order to set the quadratic polynomial equations for each response, a stepwise regression analysis with a backward elimination subroutine supposing automatic elimination of insignificant terms was applied to the experimental data obtained (only the significant terms with *p* < 0.05 were kept). To confirm the adequacy of the reduced quadratic models, the correlation (R) and determination (R^2^) coefficients were assessed and the analysis of variance (ANOVA) and residual analysis were performed. Three-dimensional response surface graphs were further plotted to investigate the independent variables’ effect on the system responses. The Taguchi approach, expressed as Signal/Noise ratio, was further applied to complement the optimization technique and to establish the adequate formulation factor combinations that would ensure the product’s quality and process robustness.

#### 3.2.6. Evaluation of the Collagen Sponges Performance during the Wound Healing Process

The in vivo experiment was performed on 20 Wistar male rats each weighing 180 ± 10 g. All animals used in the study were kept in standard laboratory conditions, were fed twice a day and received water *ad libitum*. The experiment was performed in compliance with the European Communities Council Directive 2010/63/UE and Law No. 43 of the Romanian Parliament from 11 April 2014 (ethic approval number 1030 from 24 May 2017).

The animals were anesthetized with ether ethylic and the hair was removed from the dorsal area. The experimental wound was induced using a special metallic device of 10 mm diameter. The device was heated in a boiling physiological serum and applied on the shaved dorsal area for 10 seconds. The severe burns measuring 10 mm diameter were sterilized and the collagen sponges were applied and fixed with a silk plaster. A control group was used in the study and the wounds were covered with sterile gauze.

The surface morphology of the wounds was recorded using a digital camera (Olympus SP-590UZ) and the wound diameter was measured every two days for 21 days. Any aspects of inflammation or infection of the wound, as well as any change in the animal’s health status were also monitored.

Statistical analyses were performed using the GraphPad Prism 6 software. Error bars reported within the charts denote the standard errors of the mean (*n* = 5). The experimental data were evaluated using the student’s *t*-test and analyses of variance. The results were considered significant at *p* < 0.05.

## 4. Conclusions

Collagen spongious matrices loaded with flufenamic acid intended to reduce the progression of burn lesions were designed according to the hypothesis that drug release at the affected site could be controlled both by the degree of sponge swelling and by drug diffusion through the swollen polymeric matrix, as well as by the topical support degradation rate.

The analysis and optimization of sponge formulations were performed based on a Box-Behnken experimental design and response surface methodology, followed by the Taguchi technique to select the formulations which were the most stable, robust and insensitive to noise factors. The formulations selected were three flufenamic acid sponges with the following ratio between collagen (%), dextran (%) and glutaraldehyde (%): 0.8:1.2:0.006 (M3), 1.2:0.6:0.012 (M8) and 1.0:1.2:0.000 (M10), the drug concentration of 0.5% being correlated with collagen dry substance.

The treatment of experimental burns on animals with the above selected sponges accelerated the wound healing process and promoted faster regeneration of the affected epithelial tissues compared to the control group. Further biological studies will be necessary to elucidate the mechanism of action in the tissue restoration stage of the burn healing process.

## Figures and Tables

**Table 1:** Process variables and experimental conditions in 3-factor, 3-level Box-Behnken experimental design.

Independent Variables	Coded Symbol	Coded and Uncoded Variation Levels		
		Low (1)	Middle (2)	High (3)
Collagen, C (g%)	X_1_	0.8	1.0	1.2
Dextran, DX (g%) *	X_2_	0.0	0.6	1.2
Glutaraldehyde, GA (g%) *	X_3_	0.0	0.0060	0.012
**Dependent Variables**	**Coded Symbol**	**Constraints**		
Swelling ratio, SR (g/g)	Y_1_	Maximize		
Released percent, RP (%)	Y_2_	Maximize		
Weight loss, WL (%)	Y_3_	Minimize		

* the amounts of DX and GA are reported to collagen dry substance.

**Table 2:** Formulation factors values in different Box-Behnken experimental trials for the hydrogels used to prepare the corresponding spongious matrices; observed and predicted responses for the spongious matrices.

Trials No.	Exp. Name	Independent Variables (Coded Level/Physical Level)			Responses					
		X_1_—C	X_2_—DX	X_3_—GA	Y_1_—SR (g/g)		Y_2_—RP (%)		Y_3_—WL (%)	
					Obs.	Pred.	Obs.	Pred.	Obs.	Pred.
1	M1	1 (0.8)	1 (0.0)	2 (0.006)	45.65	45.81	75.13	75.41	38.02	36.77
2	M2	3 (1.2)	1 (0.0)	2 (0.006)	28.41	28.91	76.51	75.60	32.49	31.37
3	M3	1 (0.8)	3 (1.2)	2 (0.006)	40.14	39.81	86.81	87.43	34.20	33.54
4	M4	3 (1.2)	3 (1.2)	2 (0.006)	36.16	36.21	87.28	87.62	26.62	26.53
5	M5	1 (0.8)	2 (0.6)	1 (0.000)	38.77	39.34	88.38	87.65	36.42	37.21
6	M6	3 (1.2)	2 (0.6)	1 (0.000)	26.52	27.24	84.81	85.66	30.66	31.01
7	M7	1 (0.8)	2 (0.6)	3 (0.012)	31.56	31.34	79.12	78.94	27.32	28.97
8	M8	3 (1.2)	2 (0.6)	3 (0.012)	24.05	22.94	81.61	81.31	21.48	22.77
9	M9	2 (1.0)	1 (0.0)	1 (0.000)	37.23	36.12	83.38	82.11	35.36	36.40
10	M10	2 (1.0)	3 (1.2)	1 (0.000)	42.88	42.35	93.51	94.13	31.56	32.37
11	M11	2 (1.0)	1 (0.0)	3 (0.012)	35.13	35.55	73.69	75.58	28.40	28.15
12	M12	2 (1.0)	3 (1.2)	3 (0.012)	29.83	30.62	89.19	87.60	25.83	24.12
13	M13	2 (1.0)	2 (0.6)	2 (0.006)	33.59	33.61	95.09	95.43	33.24	32.32

**Table 3:** Determination coefficients for flufenamic acid release from collagen sponges determined by application of Higuchi and Power law models; kinetic parameters for Power law model.

Formulation	Higuchi Model	Power Law Model	Release Exponent	Kinetic Constant (1/min^n^)
M1	0.9296	0.9727	0.33	0.094
M2	0.9698	0.9803	0.41	0.056
M3	0.9452	0.9730	0.37	0.089
M4	0.9206	0.9577	0.36	0.099
M5	0.9679	0.9945	0.35	0.095
M6	0.9739	0.9830	0.42	0.062
M7	0.9006	0.9531	0.33	0.108
M8	0.9355	0.9618	0.38	0.079
M9	0.9851	0.9932	0.41	0.060
M10	0.9637	0.9779	0.40	0.077
M11	0.9672	0.9807	0.41	0.057
M12	0.9659	0.9822	0.39	0.076
M13	0.9375	0.9740	0.34	0.110

**Table 4:** Analysis of variance (ANOVA) for reduced regression polynomial models.

Responses	Sources of Variation	Sum of Squares	df	Mean Squares	*F*-Value	*p*-Value
Y_1_	Regression	491.473	7	70.210 0.963	72.862	0.000099 <0.001
	Residual	4.8180	5			
	Total	496.291	12			
Y_2_	Regression	538.351	6	89.725 1.834	48.907	0.000078 <0.001
	Residual	11.007	6			
	Total	549.358	12			
Y_3_	Regression	258.315	3	86.105 1.550	55.54	0.000004 <0.001
	Residual	13.953	9			
	Total	272.268	12			

**Table 5:** Optimal combinations of independent variables coded levels, their effect size on S/N ratio for the dependent variables, expected and observed S/N value.

Control Factors (Independent Variables)	Y_1_		Y_2_		Y_3_	
	“Larger—the—Better”	Effect Size	“Larger—the—Better”	Effect Size	“Smaller—the—Better”	Effect Size
X_1_	1	1.112	2	0.272	3	0.918
X_2_	3	0.702	3	0.523	2	0.343
X_3_	2	0.563	1	0.354	3	1.526
**S/N ratio expected (dB)**	33.018		39.629		−26.909	
**S/N ratio observed (dB)**	32.072		39.417		−26.641	

**Table 6:** Composition of optimal formulations and the observed and predicted values of response variables.

Spongious Matrices	Composition of Optimal Formulation	Response Variable	Observed Value	Predictive Value	Predicted Error (%)
	X_1_:X_2_:X_3_				
	C:DX:GA				
	(g%:g%:g%)				
M3	0.8:1.2:0.006	Y_1_ (g/g)	40.14	39.81	+0.82
		Y_2_ (%)	86.81	87.43	−0.71
		Y_3_ (%)	34.20	33.54	+1.97
M8	1.2:0.6:0.012	Y_1_ (g/g)	24.05	22.94	+4.84
		Y_2_ (%)	81.61	81.31	+0.37
		Y_3_ (%)	21.48	22.77	−5.67
M10	1.0:1.2:0.000	Y_1_ (g/g)	42.88	42.35	+1.25
		Y_2_ (%)	93.51	94.13	−0.65
		Y_3_ (%)	31.56	32.37	−2.50

**Table 7:** Evolution of the wound diameter (mm) after treatment with collagen sponges (M3, M8, M10).

Wound Diameter (mm)	M3 Group		M8 Group		M10 Group		Control Group	
	Mean	SEM	Mean	SEM	Mean	SEM	Mean	SEM
Day 1	10	0	10	0	10	0	10	0
Day 5	6.4 *	1.03	6.8 **	0.58	7.4 **	0.51	9.4	0.24
Day 7	6 **	0.89	6.8 **	0.37	7.4 ***	0.24	9.2	0.20
Day 10	5.4 **	0.51	6.8 *	0.58	7 *	0.32	8.2	0.37
Day 13	1.8 *	0.80	3.4	1.40	2.2	1.36	5.6	0.24
Day 15	1.4 *	0.87	0	0	0	0	1.6	0.98
Day 17	0	0	0	0	0	0	0.6	0.60
Day 21	0	0	0	0	0	0	0	0

SEM = standard error of the mean, student’s *t*-test (vs. Control) * *p* < 0.05, ** *p* < 0.01, *** *p* < 0.001.

## Abbreviations

The following abbreviations are used in this manuscript:

FFA	Flufenamic acid
NSAID	Non-steroidal anti-inflammatory drug
DDS	drug delivery systems
C	collagen
DX	dextran
GA	glutaraldehyde
SR	swelling ratio
RP	released percent
WL	weight loss
